# Carbon dots derived from algae as H_2_O_2_ sensors: the importance of nutrients in biomass†

**DOI:** 10.1039/c9na00049f

**Published:** 2019-04-09

**Authors:** Jing Zhang, Xiaojing Liu, Jun Zhou, Xuejiao Huang, Deti Xie, Jiupai Ni, Chengsheng Ni

**Affiliations:** College of Resources and Environment, Southwest University Chongqing 400716 China nijiupai@163.com nichengsheg@swu.edu.cn; Center of Nanomaterials for Renewable Energy, State Key Laboratory of Electrical Insulation and Power Equipment, Xi'an Jiaotong University Xi'an 710049 People's Republic of China

## Abstract

Carbon dots produced hydrothermally from algae were used directly for H_2_O_2_ sensing. The mineral nutrients in biomass were found be important for the composition, crystallinity, dispersion and photoluminescence (PL) quenching of the carbon dots under reactive oxygen species, which catalysed the oxidation of passivating ligands.

## Abstract 2

Effect of mineral nutrients in biomass was found to be crucial in the synthesized carbon dots, affecting their dispersibility, PL stability and crystallinity.

## Graphical abstract

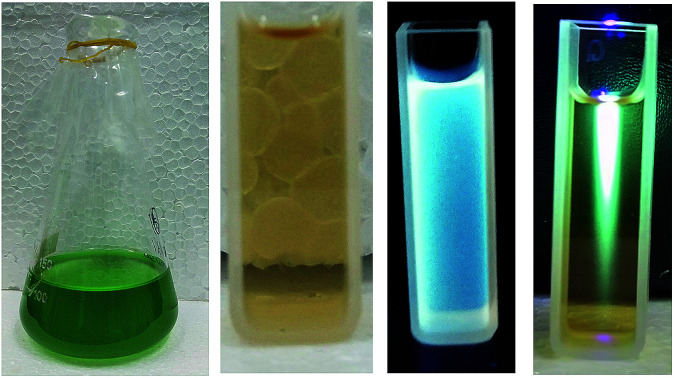

Recently, nanoscale carbon particles (denoted as “carbon dots”) with PL have been generally described as surface-passivated small carbon nanoparticles showing bandgap-like fluorescence.^1,2^ Compared to their inorganic light-emitting semiconducting counterparts, carbon dots exhibit stable fluorescence and environmental benignity (non-toxic).^1,3^ Unlike CdS or CdSe quantum dots, which are normally toxic to biological cells and aquatic systems, carbon dots show low cytotoxicity and can be used for cell imaging,^4^ and hydrogen peroxide^5–9^ and heavy metal-ion sensing.^10–12^

Carbon dots can be synthesized through the direct treatment of carbon-containing materials,^13–16^ such as carbon black,^17^ soot and Chinese inks,^18^ but the large-scale and green production of carbon dots can be achieved using biomass due to its low cost. Carbon dots have been reported to be processed directly from solid plant remains^19^ (pomelo peel,^20^ starch,^21^ lignin,^22,23^ cabbage,^24^ plant leaves,^25,26^ willow bark,^27^ Nescafe instant coffee,^28^ pipe tobacco,^29^ naked oats,^30^*etc.*), hair,^31^ urine,^12^ eggs^32^ and liquid plant extractions.^10^ The direct synthesis of carbon dots from biomass involves pre-treatment to reduce the size of the biomass using a physical method or carbonization/dehydration through pyrolysis or acidic treatment^33^ depending on the physical and chemical state of the renewable feedstock.

The use of algae as a carbon resource is sustainable as a paradigm for the reclamation of wastes, and it can address the accumulation of waste and provide an economical route compared with the use of industrial chemicals. Due to the small size and the adverse ecological effect of microalgae in degrading water quality and aquatic living beings, Ramanan *et al.*^34^ produced large quantities of carbon dots using these algae mixed with phosphoric acid solution under thermal treatment in a microwave, where nearly no prior pre-treatment was needed on the algae to achieve large-scale production. The biological nature of the carbon source affects the actual elemental composition in the final product. For example, the amino acids in the protein will act as a nitrogen source, while the existence of chlorophyll would contribute to Mg/N in the final product besides carbon. Likewise, there are also essential mineral elements, such as K, Na, P, and Fe, in the biomass,^35^ which can be transferred to the final suspension of carbon dots. Due to the presence of these elements in biomass, the resultant carbon dots are not pure carbon. However, the effect of these mineral nutrients on the properties of carbon dots has been neglected to the best of our knowledge.

Many previous reports illustrated carbon dots comprised of a carbonized core, and the crystalline graphitic units were regarded as the universal structure, but the graphitization was not always prominent due to the doping of N, S, H and O atoms in the structure.^36^ The XRD pattern (Fig. S1†) of the solid-state carbon dots from spirulina after hydrothermal treatment at 180 °C (experimental details in ESI†) shows a wide and broad peak at a 2*θ* value of 21.56°, which is attributed to the highly disordered carbon atoms, and the corresponding *d*-spacing of 0.41 nm is larger than the graphite (002) lattice spacings. The elemental analysis of the solid product (Fig. 1a) shows N 8.99%, C 43.80%, H 6.06% and S 1.02% in weight percentage. Additionally, 40.12 wt% of the material is not accounted for by the above four elements, which can be attributed to the oxygen in organic bonding or the inorganic materials as of oxides or salts. The thermogravimetric analysis (TGA) curve under a synthetic air gas (Fig. S2†) gives the overall estimation of the inorganic materials in the carbon dots, and it can be seen that approximately 14 wt% material was left behind even after the thermal treatment at 800 °C. The oxygen in the organic bonding was calculated to be 26 wt%, but the organic material was still carbon rich. There was a slight enrichment of minerals in the final carbon dots during the hydrothermal process since the mineral content in the raw spirulina from different places around the world^37^ was calculated to be around 10 wt% of the dry weight, which is consistent with the TGA result (10.78 wt% residue for spirulina). According to the inductively coupled plasma optical emission spectrometry (ICP-OES) (Fig. 1b), there was, but not limited to, quite a large amount of K, Mg, Ca and Cu in the final carbon dots and the ionic strength was estimated to be 0.97 mM according to the linear relation^38^ to the conductivity of the suspension, 0.97 mS cm^−1^. The UV-vis spectrum of the solid material (Fig. S3†) after evaporation of the solvent and volatile species showed a weak absorption at 1100 nm, which is consistent with the infrared absorption of carbon dots. X-ray photoemission spectroscopy (XPS, Fig. S4†) of the dried carbon dots showed obvious peaks of C1s, O1s and N1s, indicating the presence of –C=C–, C=O, C–OH, C–NH_2_, N–C=N and –C≡N after the analysis of binding energy.^39^ It should be noted that the O1s peak at 529.2 eV proves the binding between oxygen and metal cations,^40^ which is in accordance with the TGA result.

**Fig. 1:**
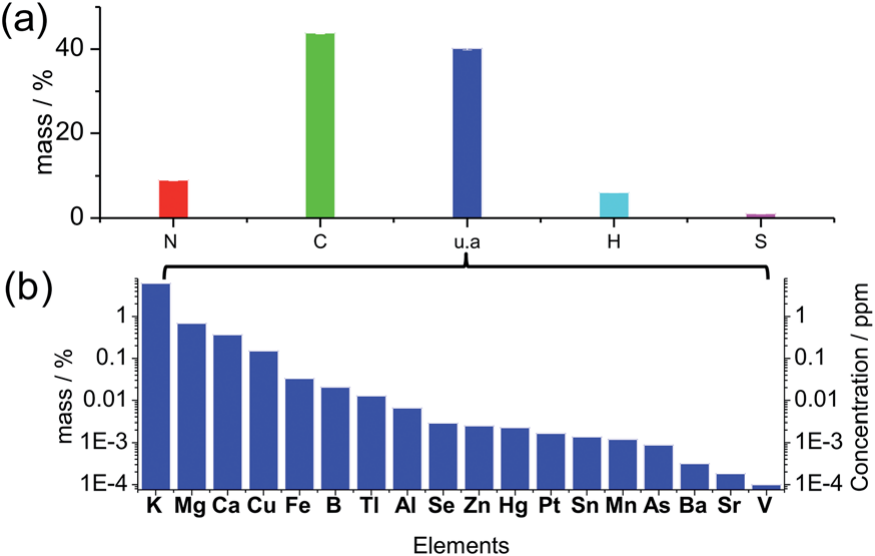
(a) Elemental analysis in terms of C, H, N and S. u.a represents the residual weight besides the above four elements. (b) Weight percentage of the measured elements in solid carbon dots (left *y*-ordinate) and concentration of the respective element in the suspension (right *y*-ordinate).

The dispersibility of carbon dots in solvents plays a significant role in their properties,^41^ which is related to the terminals and charges passivating the carbon dots. The morphology of the carbon dots was examined using scanning and transmitting electron microscopy (TEM and SEM) (Fig. 2 and S5†), where the carbon dots were on the scale of 20–50 nm and the crystallinity of the carbon dots was poor due to the doping with S, N and possibly P from the biomass. In the TEM image, the carbon dots aggregated, and a network surrounding the carbon dots evolved after ageing for eight months. The particle size analysis (PSA, Fig. S6†) on the suspension after the eight-month ageing showed that the solids were around 320 nm in size. The size from the PSA is normally larger than that from microscopy since it measures the hydrodynamic diameter of the carbon dots containing hydrophilic functional groups on their surface.^42^ However, the high salinity in this study neutralized the surface charge and destabilized the colloid system of carbon dots, and thus the size from PSA was 6 times that from microscopy. Moreover, the transition metal ion from the mineral nutrients such as Fe^3+/2+^ cations could stimulate the flocculation process.^43^ The development of aggregation and flocculation was further studied using atomic force microscopy (AFM) (Fig. 2). Similar spherical carbon dots (20–50 nm) can be distinguished on an Si single crystal; however, the flocs connecting the dots were absent in the SEM images due to the use of a carbon paper substrate. The flocs were 100 nm in length and showed excellent adhesion to the particles.

**Fig. 2:**
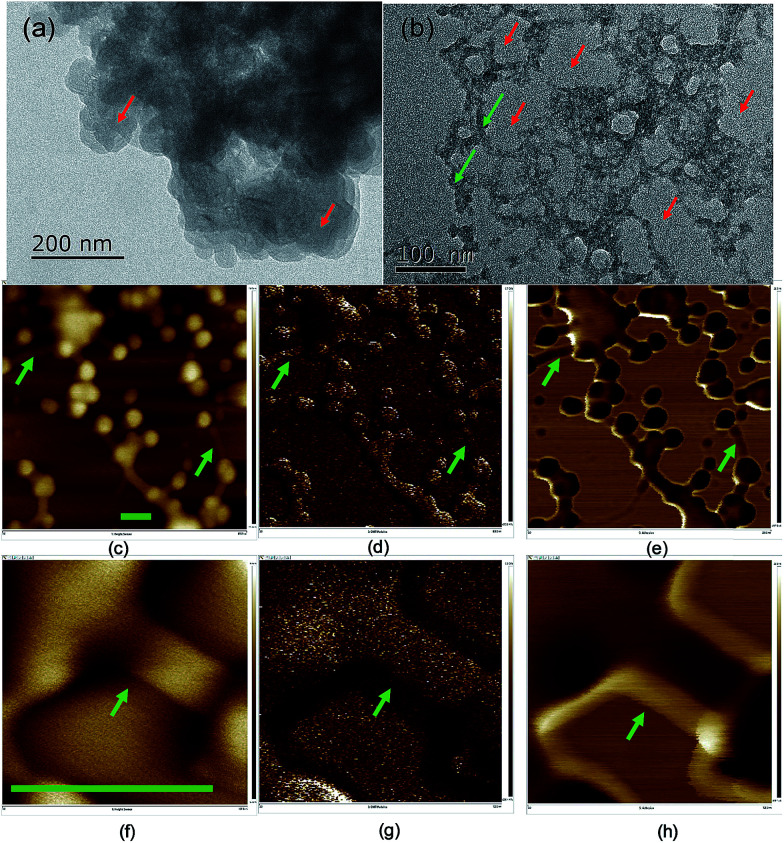
TEM images of the carbon dots before (a) and after (b) ageing. AFM images of the carbon dots on a silicon single crystal after eight months of ageing in terms of height (c, f), Young's modulus (d, g) and adhesion (e, h) at different magnifications. The scale bars in (c–h) represent 100 nm and the green arrows indicate the flocs binding the carbon dots while the red arrows indicate the carbon dots. (f–h) are images highlighting the morphology of the flocs connecting the particles.

The yellow suspension of the carbon dots showed strong blue fluorescence under a flash light of 365 nm (Fig. 3a–c). The light absorption of the suspension was measured after diluting the initial suspension by a factor of 10 to avoid the light-scattering effect.^40^ The suspension showed a slight absorption (Fig. 3d) even at a wavelength of 700 nm and a strong absorption was evidenced at a wavelength shorter than 400 nm. A single emission peak at 440 nm stretching from 400 to 650 nm was observed when the suspension was excited at 360 nm. Moreover, the emission of the suspension was dependent on the excitation wavelength and a weak emission was found even at an excitation of 510 nm (Fig. 3d and e). The PL excitation (PLE) technique allowed the measurement of all the allowed sub-band absorption transitions,^44^ and the PLE (Fig. 3f) of the suspension with a detection wavelength, *λ*_det_, at 437 nm or 467 nm showed an absorption peak at around 350 nm. Also, the PLE peak blue shifted when the emission wavelength decreased, which is in agreement with the PL shift under different excitation wavelengths. This PLE peak is attributed to the n–π* transition of –C=O, C–N or –C–OH bonds in the sp^3^ hybridized domains, which originated from the carboxyl (–COOH), hydroxyl or amine (–NH_2_) groups on the C-dot surface.^45^ This PLE peak is in accordance with the parasitic peak at a similar position in the UV-vis light-absorption. It is known that the π–π* transition of the aromatic –C=C– under sp^2^ hybridization shows an absorption peak at 250 nm,^45,46^ but no PLE peak was found in this region, indicating that sp^3^ hybridized bonds are responsible for the fluorescence. The clear difference in the PLE spectra for *λ*_det_ at 437 and 467 nm and the PL variation upon the change of excitation wavelength can be due to the size variation of the carbon dots^47^ or the functional moieties on the surface of the carbon dots, acting as energy traps for different emissions.^44,48^ During large-scaled synthesis, coherence and reproducibility are critical for practical applications.^10^ Thus, three types of algae, including red algae (Rhodophyta), green algae (Chlorophyta) and spirulina (Cyanophyta), were used as the biomass and their PL emissions were quite similar (Fig. S7†), indicating that the algae were interchangeable as biomass for the synthesis of carbon dots. According to the elemental analysis, the N wt%/(C + N + H) wt% is 0.15, which is very close to that in protein (0.16), indicating that the protein in algae biomass is the main component in the carbon dot suspension using our synthetic temperature.^49^ For the carbon dots synthesized from lignocellulose, the lignin nanoparticles are around 100 nm in size and contain more carbon than the smaller dots.^23^ Although the composition of the three types of algae may be different, their similarity in protein rendered similar PL emissions in the carbon dots from the three types of algae. For the carbon dots derived from biomass using the hydrothermal method, the PL quantum yields (PLQYs) range from 2.6% to 21.7%, depending on the source of the biomass and processing parameters.^21,34^ The PLQY of the carbon dot suspension from spirulina at a excitation wavelength of 360 nm was calculated to be 2.86% after eight months aging according to the comparison with quinolone sulphate in 0.1 M H_2_SO_4_ (Fig. S8†).^50^ The quantum yield of the carbon dots from algae was not very high, which can be related to the size of the carbon dots or their surface moieties, but they showed remarkable stability since this value decreased by only 15% after the during the eight months from the initial 3.3%. The highly stable PLQY stability is beneficial for consistency and operation cost. Although high photoluminescence is desirable for carbon dots, but their stability against the background such as ionic strength and pH is also very important for their practical use.^51^

**Fig. 3:**
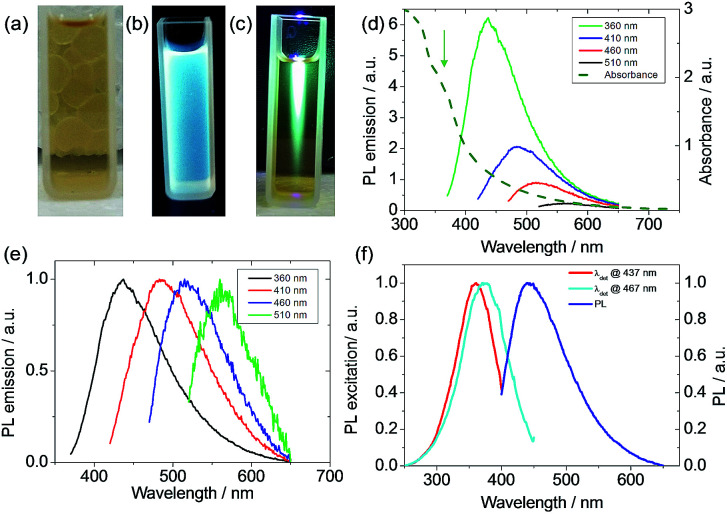
Image of the suspension (a), and illuminated under a 365 nm flash light (b) and 365 nm light beam (c). (d) Absorbance and PL emission spectra under different excitation wavelengths. The arrow shows the shoulder peak at 350 nm in the absorption spectra. (e) Normalized PL emission to highlight the peak positions. (f) PLE of the suspension with detection wavelengths at 437 and 467 nm.

The carbon dots showed stable PL (Fig. 4) when [KCl] changed from 10^−9^ to 10^−2^ M and in the pH range from 3 to 9. The pH-dependence of the PL of carbon dots is normally related to the protonation/deprotonation of the functional groups in their passivating species,^52^ and the stability of the PL against pH implies the low acid-dissociation constant, such as in alcohols. The quenching of Co^2+^, Cr^2+^ and Fe^3+^ was measured against the concentration of the respective ions, and a linear change was observed between *I*_0_/*I* (where *I*_0_ and *I* are the intensities of PL at 440 nm without and with quencher, respectively) and [Fe^3+^], but no discernible difference was found between Cr^3+^ and Co^2+^ cations as PL quenchers. This indicates that these carbon dots are more sensitive to Fe^3+^ than the other two cations. The PL quenching of cations on passivated (N-doped-) carbon dots is normally attributed to the strong binding between the cation and the hydroxyl group, which creates an additional pathway of hot charge carriers from the carbon dots under illumination.^46,53^

**Fig. 4:**
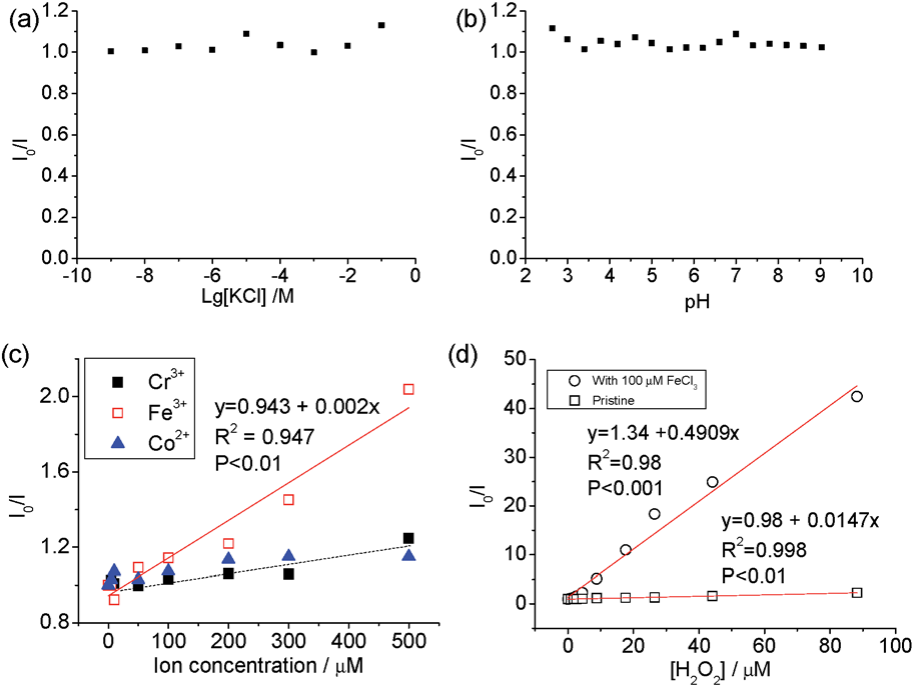
PL quenching (*I*_0_/*I*) with different KCl concentrations (a), pH (b), concentration of Cr^3+^, Fe^3+^ and Co^2+^ (c), and H_2_O_2_ (d). (d) shows the plots of *I*_0_/*I* against [H_2_O_2_] with and without Fe^3+^ as co-quencher.

Carbon dots produced from pure chemicals are relatively stable under H_2_O_2_ alone as a PL quencher.^54–56^ Boron- and silicon-doped carbon dots^6,7^ were used for the detection of H_2_O_2_, and B and Si were reported to be the active sites. However, the carbon dots derived from biomass with transition metal cations exhibited superior results. Specifically, good linearity (*χ*^2^ = 0.998) was observed in the plot of *I*_0_/*I* against [H_2_O_2_] and the slope of the line, *K*_sv_, was calculated to be 14.7 × 10^3^ M^−1^, which is seven times higher than that when Fe^3+^ was used as the quencher (2 × 10^3^ M^−1^). The variation in *K*_sv_ and linearity indicates that carbon dots with mineral nutrients are much more suitable for H_2_O_2_ sensing than that metallic ions.

As reported previously, the simultaneous addition of Fe^3+^ and H_2_O_2_ to carbon dots in acidic conditions induced more significant PL quenching than either agent alone.^54,55^ The Fenton reaction-meditated detection of H_2_O_2_ was proposed when Au nanoparticles passivated by *N*-acetyl-l-cysteine were used for the detection of H_2_O_2_,^57^ but carbon dots with metal cations also undergo similar PL quenching.^55^ Qian *et al.*^58^ added 1 μM Fe^2+^ to a suspension of N-doped carbon dots and good sensitivity was achieved in the concentration range of 0 and 1 μM H_2_O_2_. The *K*_sv_ for the samples with double quenchers, 100 μM Fe^3+^ and H_2_O_2_, was 33 and 245 times higher than that with H_2_O_2_ or Fe^3+^ only, respectively, indicating that the Fenton reaction accelerates the oxidation of organic material, and thus plays an important role in the emission property of carbon dots with a high transition metal cation concentration. The addition of 100 μM Fe^3+^ decreased the linearity of the PL quenching, indicating that the amount of minerals in the biomass was sufficient to promote PL quenching for sensing. As indicated in Fig. 1b, transition metal cations (*e.g.* [Fe^3+/2+^] + [Cu^2+/1+^] = 0.1 mg kg^−1^ or 1.6 μM considering the dilution coefficient of 10) were present in the algae-based carbon dots, which affected the generation of oxidizing species from H_2_O_2_ and the passivation of the carbon dots.

The ^1^H nuclear magnetic resonance (NMR, Fig. S9†) study confirmed that the possible mechanism of H_2_O_2_ sensing by the carbon dots may be related to the change in passivation due to the catalysis of the oxidation process by the metal ions from the mineral nutrients in the algae. The NMR spectra of the suspension with H_2_O_2_ clearly indicate the formation of carboxylic acid, where the NMR peaks of acetic acid, as an example, are located at 11.42 and 2.098 ppm for the proton in –COOH and α-proton on the methyl group, respectively.^59^ Since carboxylic acid is more electrophilic than alcohols,^60^ it will be a better PL quencher in carbon dots. Since the emission wavelength of the carbon dots is strongly dependent on the passivating ligands and in-gap states,^44^ and the conversion of –OH or ester into –COOH was confirmed *via* NMR, one would expect a change in the emission spectra of the carbon dots with different quenchers. The PL of the carbon dots (Fig. 5) could be fitted with two Gaussian peaks at 428 nm and 477 nm. It is clear that the increase in oxidation state due to the addition of Fe^3+^ and H_2_O_2_ as a co-quencher enhanced the emission at a longer wavelength, which is in accordance with the previous study on the surface oxidation of carbon dots.^48,61^ Also, if [H_2_O_2_] is higher than 17.6 μM, the emission at 477 nm is dominant due to catalysis of the oxidation of surface moieties (Fig. S10†) by the 100 μM Fe^3+^. The addition of Fe^3+^ alone does not change the area ratio between the peaks at 477 and 428 nm, *A*_477 nm_/*A*_428 nm_, which is consistent with the small *K*_sv_ value and poor linearity for Fe^3+^ sensing, while the addition of H_2_O_2_ steadily increased the *A*_477 nm_/*A*_428 nm_ ratio, which is related to the small amount of metallic ions from the nutrients in the plant since carbon dots from pure carbon sources are stable against H_2_O_2_.

**Fig. 5:**
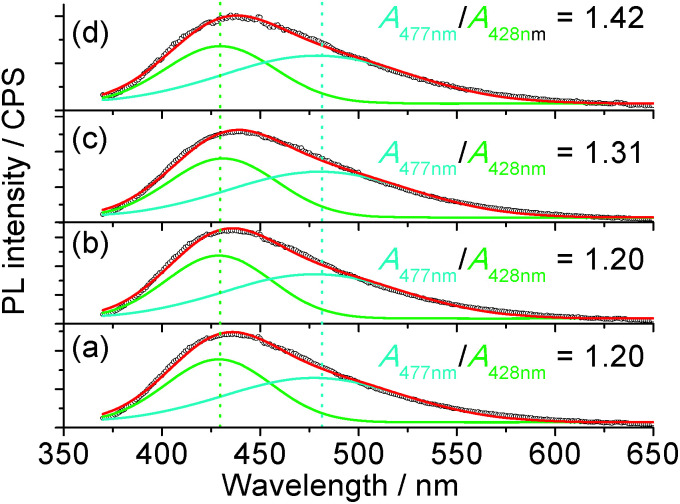
Representative PL spectra of the pristine carbon dots (a), with 100 μM Fe^3+^ (b), 88 μM H_2_O_2_ (c) and 100 μM Fe^3+^ plus 4.4 μM H_2_O_2_ (d). The excitation wavelength was 360 nm and *A*_477 nm_/*A*_428 nm_ indicates the ratio of the area between the peaks at 477 nm and 428 nm. The *R*^2^ value of the fitting of the curves was 0.996.

In summary, algal biomass was utilized to produce carbon dots for blue light-emitting sensors and the effect of the residual mineral nutrients in the biomass was studied as an important parameter for PL upon the addition of H_2_O_2_. This method can be used for mass production since the PL emission of the carbon dots barely changed when they were obtained from different subgroups of algae. The carbon dots of 30 to 50 nm in size were N- and S-doped carbon dots and mixed with around 10 wt% mineral nutrients. The minerals in the suspension destabilized the colloid *via* precipitation and flocculation after lengthy ageing, but showed a limited effect on the PLQY of the suspension. The sensitivity and linearity of the PL quenching are related to the passivating species, where the PL quenching of the carbon dots in this study under H_2_O_2_ is related to the oxidation of the passivating species on the surface to carboxylate due to the Fenton reactions involving the cations of transition metals in the biomass. This study is beneficial for understanding the impact of mineral nutrients on the colloids of suspensions and the selection of biomass for mass production and sustainable synthesis of carbon dots.

## Conflicts of interest

There are no conflicts of interest to declare.

## Supplementary Material

NA-001-C9NA00049F-s001
