# Supplementary material for: Carbon dots derived from algae as H2O2 sensors: the importance of nutrients in biomass
Source: Nanoscale Adv. 2019 Apr 9;1(6):2151–6. doi: 10.1039/c9na00049f (PMC9419849; doi:10.1039/c9na00049f)
Supplement: NA-001-C9NA00049F-s001 [file NA-001-C9NA00049F-s001.pdf]

Electronic Supplementary Material (ESI) for Nanoscale Advances.
This journal is © The Royal Society of Chemistry 2019

Supplementary Materials

Experimental

Three types of algae (red algae (*Rhodophyta*), green algae (*Chlorophyta*) and spirulina (*Cyanophyta*)) were used to produce carbon dots. The algae were collected in natural lakes in China and washed with denoised water and mist dried for future use. 0.1 g of algae and 75 mL deionized water was mixed to form a suspension. The admixture was sonicated for 30 seconds before being transported to a 100-mL autoclave with polytetrafluoroethylene lining which was then heated in an oven at 180 ^{o}C for 10 hours. The heating and cooling rate of the oven was 1.67 ^{o}C/minutes. Large particles after the hydrothermal process were removed via the filtration through a 0.45 μm filter and then the suspension was collected for future use. Elemental analysis for metallic ion was performed on the an inductively coupled plasma atomic emission spectroscopy (ICP-AES, Agilent 5110, USA) without further dilution or concentration of the colloidal suspension. The conductivity of the colloidal suspension was measured with a multimeter (Keithley 2100) equipped with a rig containing two platinum plates (0.3 cm^2). Photoluminescence (PL) of the suspension was measured after diluting the suspension by 10 times with denoised water on a superfluorimeter (FLS980, Edinburgh Instrument). The diluted suspension containing different amount of Cr^{2+}, Co^{2+}, Fe^{3+} and H_2O_2 were prepared via the addition of $[Cr(H_2O)_6](NO_3)_3$•$3H_2O$, $Co(NO_3)_2$•$6H_2O$, $FeCl_3$ and 30 wt.% H_2O_2 (all from Aladdin Chemicals, analytical reagent), respectively, and then PL was repeated after 10 hours to ensure the full equilibration, though an instant stable PL (within 5%) as an example can be obtained after the addition of H_2O_2 (Figure S11). ^{1}H nuclear magnetic resonance (^{1}H NMR) of the suspension being concentrated by 50 times in D_2O was performed on a AVANCE III spectrometer (Bruker, Germany) and the chemical shift was calibrated against tetramethyl silane (TMS). The particle size analysis (PSA) of the suspension was carried out with laser-diffraction methods based on photon cross-correlation spectroscopy (λ=658 nm, Nanophox, Sympatec GmbH, Germany) after 8 months of ageing to detect flocculation process.

The solid carbon dots were collected by drying the suspension at room temperature and then a productivity of 15 % was obtained in relation to the weight of starting algae. Elemental analysis for

C, H, N and S was performed on a pyrolysis elemental analyzer (Elementar Vario Micro Select, Elementar Analysensysteme Germany). To measure the residual materials apart from the volatile elements such as C, H, O and S, thermogravimetric analysis (TGA) was carried out on the raw spirulina and solid-state carbon dots in a synthetic air atmosphere up to 800 °C. X-ray diffraction (XRD) and diffuse reflectance spectroscopy (DRS) of the collected solids were measured at room temperature on a monochromatic X-Ray spectrometer (Cu K_{α} = 0.154 nm, XD-3, Parsee, China) and an ultraviolet-visible (UV-Vis) spectrometer equipped with a 10-cm integrating sphere (UV-1800 PC, Macy, China). Scanning electron microscopy (SEM) and transmitting electron microscopy (TEM) of the sample was examined on a JSM 7500F, JEM2100 microscope (JEOL, Japan) via the drying of the suspension aged for eight-months on a carbon paper and a copper grid, respectively. The accelerating voltage of the TEM is 200 kV. The morphologies of samples prepared by dripping the suspension on silicon substrate were further examined by atomic force microscope (AFM, Bruker Dimension Icon; operated in the peakforce QNM mode at 0.8 Hz scanning rate). Platinum–iridium-coated probes (SCM-PIT, Bruker) were used in the AFM measurements. X-ray photoelectron spectroscopy (XPS) data were obtained on a ESCALAB.250Xi spectrometer (Thermofischer Scientific, USA) using monochromatic Al Kα X-rays (1486 eV) for the overall survey and high-resolution core level of C 1s. N 1s and O 1s.

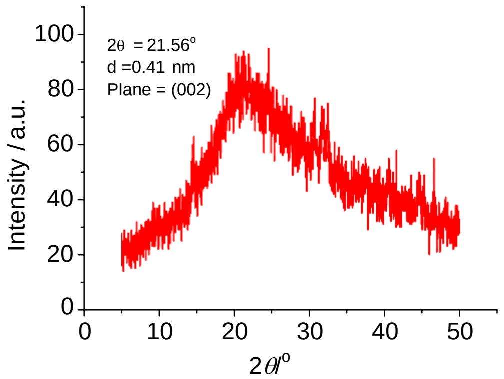

Figure S1. XRD of the dried carbon dots.

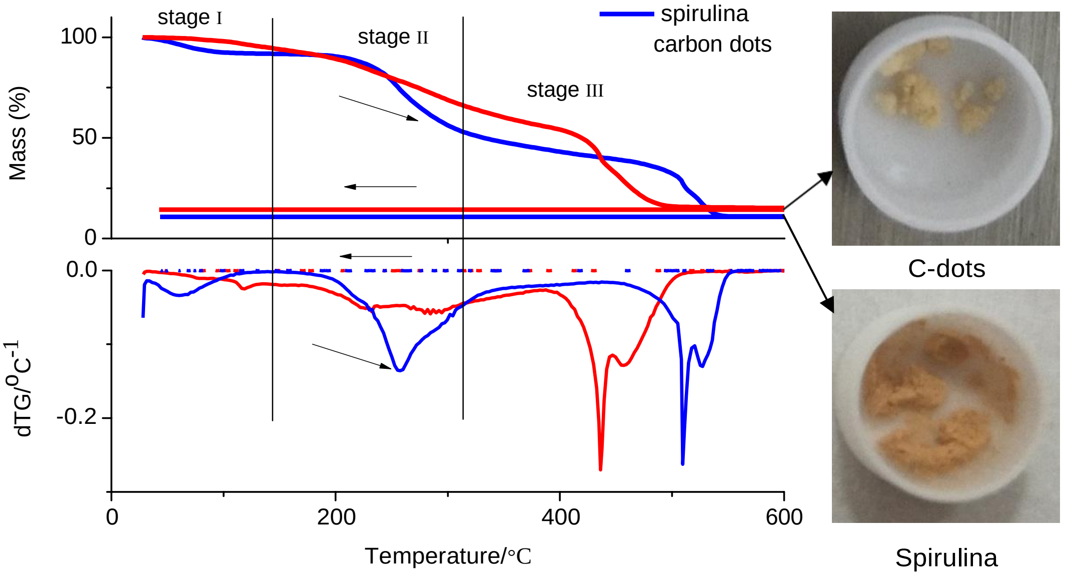

Figure S2 TGA was performed on the raw spirulina and the corresponding carbon dots after the hydrothermal process for 10 hours. It can be seen that a significant amount of ash (14 wt.% related to the initial solids) was left behind in the crucible after the thermal treatment at 800 °C, which could be related to the inorganic oxides or salts. In detail the weight loss during the heating process contains three stages: stage I (centered at 100 °C) could be related to the moist or other small molecules absorbed on the surface, stage II (centered at 260 °C) which could have linked to the alkyl chains and the final stage centered at 440 °C for carbon dots and 510 °C for spirulina could linked to the carbon related species. After the annealing in air atmosphere, the product is yellowish, indicating there could be very little carbon left in the residue.

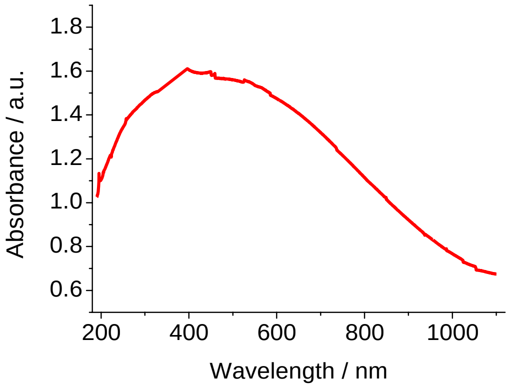

Figure S3 Absorbance of the dried carbon dots using DRS.

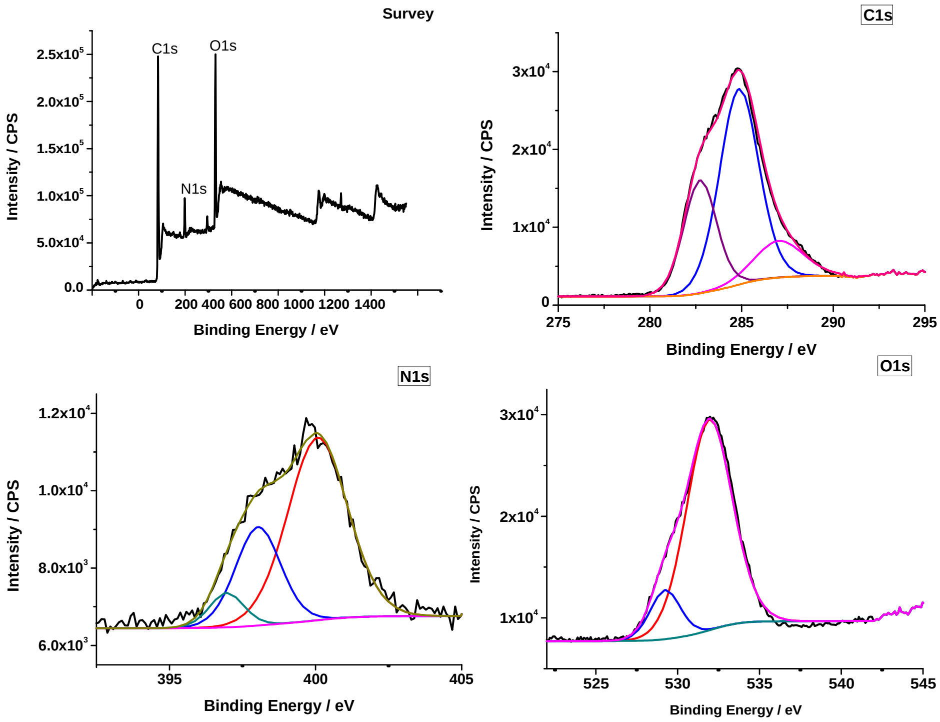

Figure S4. XPS of the carbon dots in terms of the overall survey, and detailed scan for C1s, N1s and O1s

The core level spectrum of C1s can be fitted into three peaks: 282.8,284.8and286.9 eV. The peak at 284.8 eV and 282.8 eV can be attributed to the graphitic carbon sp^3 and sp^2 hybridization, respectively. The peak at 286.9 eV could related to the C=O or C-OH bounds according to the research on graphene oxide.[1] The high-resolution spectrum for the N1s can be deconvoluted into three peaks at 400.1 eV, 398.0 eV and 396.9 eV. The strongest peak at 400.1 eV was normally related to the $C-NH_2$ [2] or $O=C-NH_2$, while the peak at 398.0 eV was normally attributed to the sp^2 hybridization nitrogen in the tri-s-triazine ring (C-N=C), while the peak at 396.9 eV corresponds to the sp hybridization nitrogen in cyanogen (-C≡N). For the high-resolution peak of O1s, two peaks centered at 532.0 and 529.2 eV can be distinguished and the first one can be attribute to the oxygen in absorbed hydroxyl group and water and oxides, respectively [3].

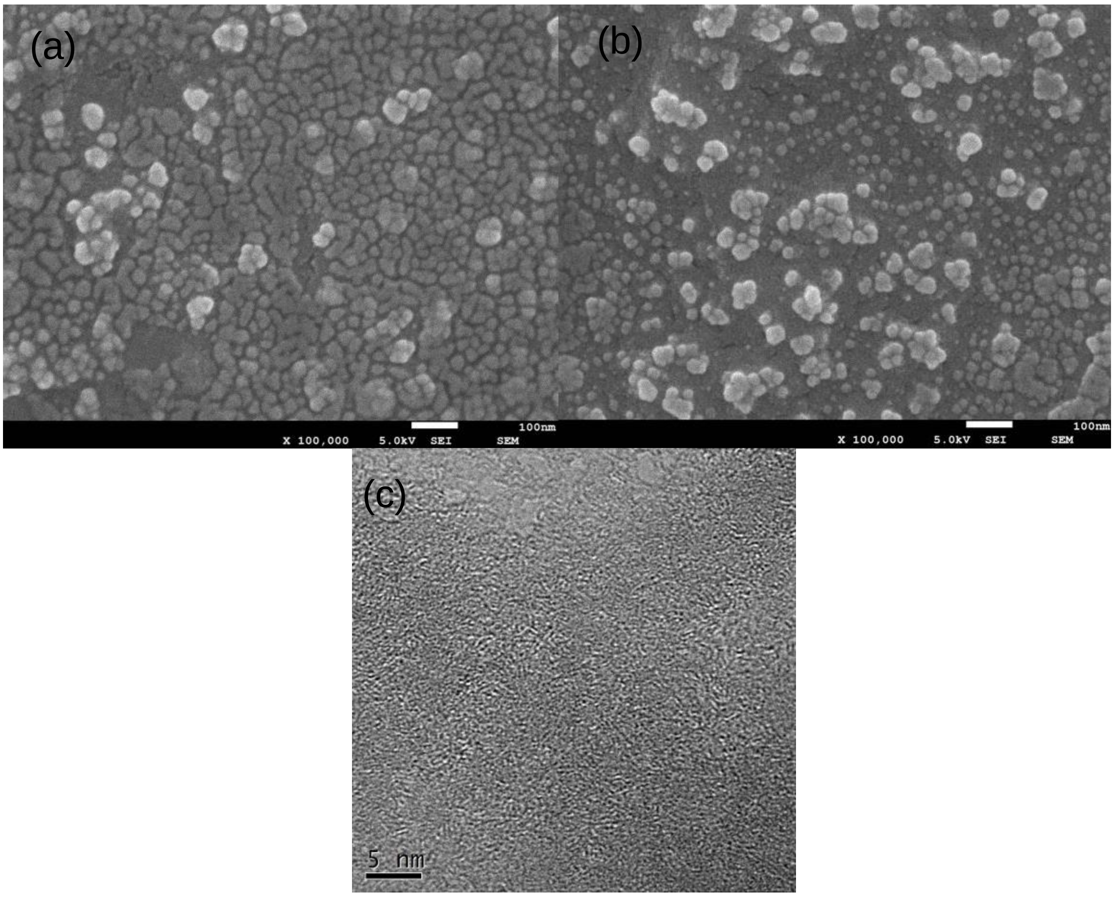

Figure S5. SEM image of the carbon dots before (a) and (b) the ageing process. (c) high-resolution TEM image of the carbon dot.

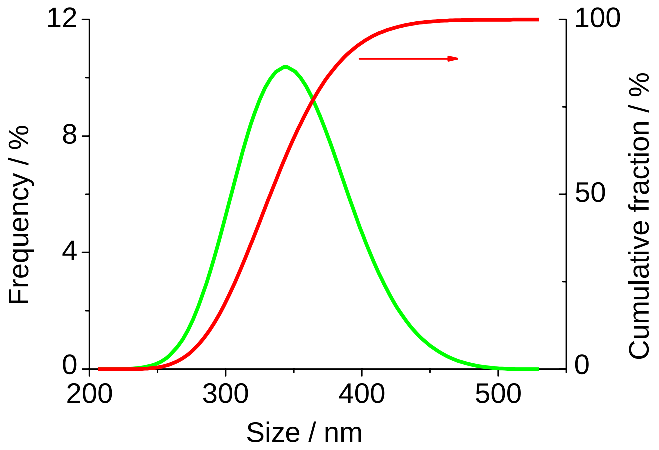

Figure S6 Particle size analysis of the carbon dots using laser-diffraction methods after eight months of ageing in colloidal suspension.

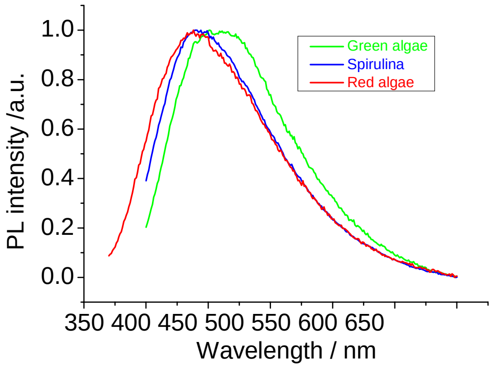

Figure S7 PL emission of the carbon dots from three different types of algae under an excitation wavelength of 360 nm.

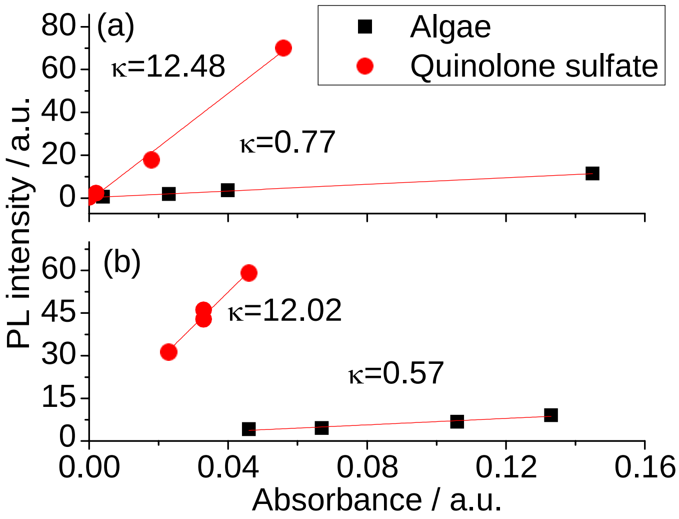

Figure S8 Plots of integrated PL intensity of fresh (a) and aged (b) carbon dots from spirulina and quinolone sulfate (referenced dye) as a function of optical absorbance at 360 nm. "κ" indicates the slope of absorbance and PL intensity. The PL quantum yield was set to be 55% and the quinolone sulfate was dissolved in 0.1 M H_2SO_4.[1] Four samples for the referenced dye and carbon dots were prepared by diluting the initial suspension for the specific absorption and PL. The quantum yield of the fresh and eight-month aged sample is 3.3 % and 2.86 %, respectively. The linear relationship between the PL and light absorption indicates that validity of the comparison.

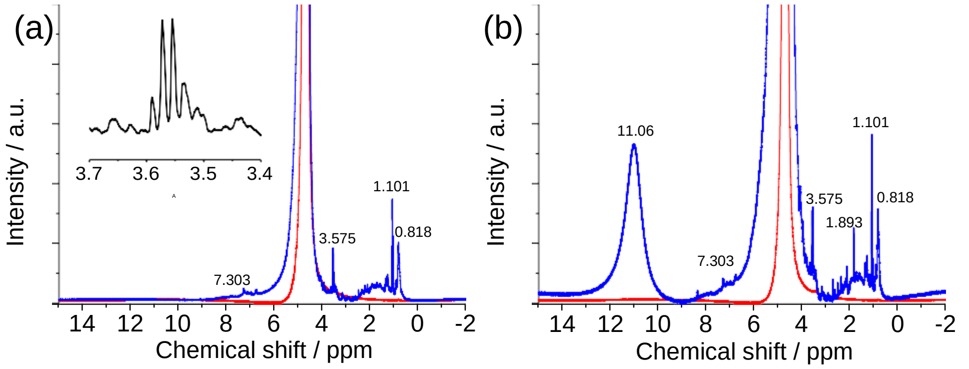

Figure S9. NMR spectra of the suspension concentrated by 5 times before (a) and after (b) the addition of H_2O_2. The red line showed the back ground and the inset in (a) showed the multiple peaks with chemical shift around 3.55 ppm. The peak at 4.79 ppm is the one for D_2O or water.

The pristine suspension was concentrated by 5 times (after considering the dilution by D_2O in the sample preparation) *via* the evaporation at room temperature for several days and the ^{1}H NMR spectra (Figure S9(a)) were obtained to show significant peaks. When the suspension is concentrated, the total metal ions will be also accumulated to reach 40 mg/kg according to the ICP results, but the concentration of Fe cation is still much lower than those of 100 μM. There is a bump in the NMR spectrum covering the range 0.5 to 3 ppm in chemical shift, δ, which could be a result of the mixing of multiple functional groups in this range. However, one can identify four distinct peaks at 7.303, 3.575, 1.101 and 0.818 ppm. The first two peak can be assigned to the proton in -CH_2- and -CH_3 respectively in an alkyl group, and this can be verified by the splits of the peaks due to the resonance with the proton on neighboring carbon[2,3]. The singlet at 0.818 ppm and the weak peak at 7.303 cannot be assigned to specific functional groups due to the complexity in the system because of N and S in the system. With the addition of 150 μM H_2O_2, two distinct peaks evolved (Figure S9(b)): a broad one at 11.06 ppm and the other at 1.893 ppm. These two peaks clearly indicate the formation of carboxylic acid as the NMR peaks of acetic acid as an example reside at 11.42 and 2.098 ppm for the proton in -COOH and methyl group, respectively[3].

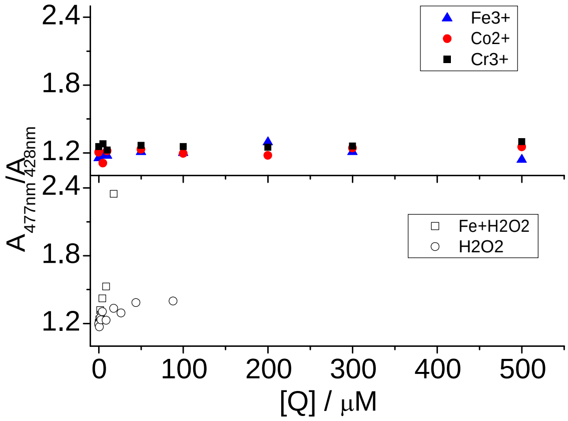

Figure S10. Concentration dependence of A_{477nm}/A_{428nm} for the different quenchers as Fe^{3+}, Co^{2+}, Cr^{3+}, H_2O_2 and H_2O_2 plus 100 mM Fe^{3+}.

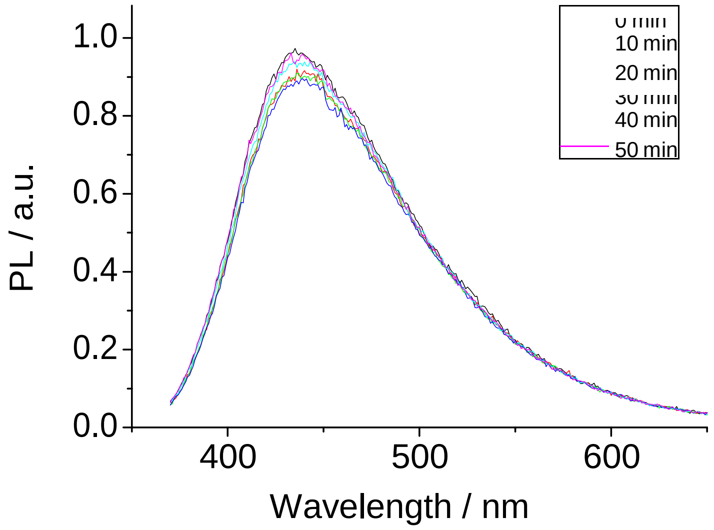

Figure S11. PL intensity of the carbon dots with 88 μM H_2O_2 at different times after the preparation of mixture.

1. H. E. Gottlieb, V. Kotlyar and A. Nudelman, J. Org. Chem., 1997, 62, 7512-7515.
2. C. Ni, D. Carolan, C. Rocks, J. Hui, Z. Fang, D. B. Padmanaban, J. Ni, D. Xie, P. Maguire, J. T.S. Irvine and D. Mariotti, Green Chem., 2018, 20, 2101-2109.
3. H.R.S. Hassan, G. Zhang, X. Wang, D. Xie, J.T.S. John, J.Ni, C. Ni,Chem. Eng. J. ,2019,15, 875-884.
